# Osteocytes/Osteoblasts Produce SAA3 to Regulate Hepatic Metabolism of Cholesterol

**DOI:** 10.1002/advs.202307818

**Published:** 2024-04-13

**Authors:** Shijiang Huang, Yuanjun Jiang, Jing Li, Linlin Mao, Zeyou Qiu, Sheng Zhang, Yuhui Jiang, Yong Liu, Wen Liu, Zhi Xiong, Wuju Zhang, Xiaolin Liu, Yue Zhang, Xiaochun Bai, Bin Guo

**Affiliations:** ^1^ State Key Laboratory of Organ Failure Research Department of Cell Biology School of Basic Medical Sciences Southern Medical University Guangzhou Guangdong 510515 China; ^2^ Department of Obstetrics and Gynecology Nanfang Hospital Southern Medical University Guangzhou Guangdong 510515 China; ^3^ Department of Biochemistry and Molecular Biology School of Basic Medical Sciences Southern Medical University Guangzhou Guangdong 510515 China; ^4^ Equipment Material Department West China Xiamen Hospital of Sichuan University Xiamen Fujian 361000 China; ^5^ Central Laboratory The Fifth Affiliated Hospital Southern Medical University Guangzhou Guangdong 510900 China; ^6^ Guangdong Provincial Key Laboratory of Bone and Joint Degenerative Diseases The Third Affiliated Hospital of Southern Medical University Guangzhou Guangdong 510630 China; ^7^ The Tenth Affiliated Hospital Southern Medical University Dongguan Guangdong 523018 China

**Keywords:** bone‐liver crosstalk, CYP7A1, hypercholesterolaemia, osteocytes/osteoblasts, SAA3

## Abstract

Hypercholesterolaemia is a systemic metabolic disease, but the role of organs other than liver in cholesterol metabolism is unappreciated. The phenotypic characterization of the *Tsc1^Dmp1^* mice reveal that genetic depletion of tuberous sclerosis complex 1 (TSC1) in osteocytes/osteoblasts (*Dmp1*‐*Cre*) triggers progressive increase in serum cholesterol level. The resulting cholesterol metabolic dysregulation is shown to be associated with upregulation and elevation of serum amyloid A3 (SAA3), a lipid metabolism related factor, in the bone and serum respectively. SAA3, elicited from the bone, bound to toll‐like receptor 4 (TLR4) on hepatocytes to phosphorylate c‐Jun, and caused impeded conversion of cholesterol to bile acids via suppression on *cholesterol 7 α‐hydroxylase* (*Cyp7a1*) expression. Ablation of *Saa3* in *Tsc1^Dmp1^* mice prevented the CYP7A1 reduction in liver and cholesterol elevation in serum. These results expand the understanding of bone function and hepatic regulation of cholesterol metabolism and uncover a potential therapeutic use of pharmacological modulation of SAA3 in hypercholesterolaemia.

## Graphical abstract

Cholesterol homeostasis requires collaboration among organs; however, the involvement of bone is unappreciated. The present work elucidates a mechanism by which bone‐derived SAA3 inhibits the expression of CYP7A1 by binding to TLR4 on hepatocytes and thereby phosphorylates c‐Jun, which leads to hypercholesterolaemia in *Tsc1^Dmp1^* mice. Together, they identify SAA3 as a novel bone‐derived factor that regulates hepatic metabolism of cholesterol.

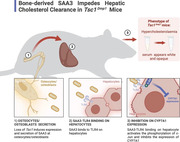

## 1 Introduction

Hypercholesterolaemia is a common systemic disease characterized by elevated low‐density lipoprotein (LDL) cholesterol level in serum and increased risk of atherosclerotic cardiovascular diseases,^[^ ^1^ ^]^ peroxisome disorders,^[^ ^2^ ^]^ Alzheimer disease,^[^ ^3^ ^]^ and cancers.^[^ ^4^ ^]^ Lifestyle changes such as regular exercises and healthy diets are the first line of defense against high cholesterol, implying a vital role for the coordination of multiple organs and tissues in the maintenance of systemic cholesterol homeostasis. Skeletal system is the primary responder to exercise stimuli. Classic theories consider the bone as the body's supportive and protective organ par excellence. Whereas, accumulating evidence now reveals that the bone is also a secretory organ, producing a variety of signaling proteins in the setting of external stimuli, and regulating skeletal muscle mass, cognitive function, glucose homeostasis, immune function,^[^ ^5^ ^]^ et al. However, roles for bone in regulating systemic cholesterol homeostasis, as well as the mechanisms participating in this process, remain obscure.

The bone encompasses osteocytes, osteoblasts, osteoclasts, and osteoprogenitor cells. Osteocytes, the most abundant type of bone cells, are revealed to serve as a major secretory source for signaling proteins. Fibroblast growth factor 23 (FGF23)^[^ ^6^ ^]^ is the first described secretory protein of bone and was found to be synthesized and released by osteocytes and osteoblasts to limit phosphate reabsorption at proximal and distal renal tubules^[^ ^7^ ^]^ and to inhibit parathyroid hormone (PTH) secretion at parathyroid gland.^[^ ^8^ ^]^ Recent studies also indicated that osteocytes could secrete signaling proteins, including sclerostin (SOST),^[^ ^9^ ^]^ prostaglandin E2 (PGE2),^[^ ^10^ ^]^ and interleukin‐19 (IL‐19),^[^ ^5c^ ^]^ to contribute to the regulation of distant organs. These secretion activities were observed to occur in response to exercise, hypoxia, or hormonal stimuli. Mechanistic target of rapamycin complex 1 (mTORC1) is the essential mediator for osteocytes and osteoblasts to respond to diverse stimuli. Our previous studies indicated that mTORC1 signaling participates in regulating proliferation, differentiation, and paracrine action of several types of bone cells, including osteocytes and osteoblasts, and coordinates the activities of bone and bone marrow microenvironment. Examples include bone resorption,^[^ ^11^ ^]^ bone sclerosis,^[^ ^12^ ^]^ megakaryopoiesis,^[^ ^13^ ^]^ angiogenesis,^[^ ^14^ ^]^ neutrophil development,^[^ ^5c^ ^]^ et al. However, a function for osteocyte/osteoblast mTORC1 signaling in regulating cholesterol metabolism has not yet been described.

Hypercholesterolaemia is commonly attributed to the dysregulation of cholesterol production and/or clearance. Liver harbors the catabolism of cholesterol to bile acids, which is the predominant breakdown pathway of cholesterol. Inappropriately suppressed or maladaptively sustained bile acids biosynthesis can dysregulate cholesterol metabolism. Recent studies indicated that fibroblast growth factor 15/19 (FGF15/19),^[^ ^15^ ^]^ interleukin‐1*β* (IL‐1*β*),^[^ ^16^ ^]^ and transforming growth factor‐*β*1 (TGF‐*β*1) ^[^ ^17^ ^]^ can cause hypercholesterolemia via reduction in rate‐limiting enzyme(s) and impediment on bile acids biosynthesis. An improved understanding of the cytokine‐mediated regulatory mechanisms of bile acids biosynthesis could identify therapeutic targets for cholesterol disorders.

Here, using *dentin matrix protein 1‐Cre* (*Dmp1^Cre^*)*/Tsc1^fl/fl^* (*Tsc1^Dmp1^*) mice, we show that genetic depletion of tuberous sclerosis complex 1 (TSC1), an upstream negative regulator of mTORC1 in osteocytes/osteoblasts, results in upregulation and secretion of serum amyloid A3 (SAA3). SAA3 binding to toll‐like receptor 4 (TLR4) stimulated the activation of c‐Jun and led to reduction in Cholesterol 7 *α*‐hydroxylase (CYP7A1) level in hepatocytes. CYP7A1 is the initial and rate‐limiting enzyme in the bile acid biosynthesis process,^[^ ^18^ ^]^ and the lack of CYP7A1 impeded the clearance of serum cholesterol, resulting in progressive hypercholesterolemia in *Tsc1^Dmp1^* mice, which was observed as early as 3 months of age. Consistent with this, whole‐body knockout of *Saa3* gene in *Tsc1^Dmp1^* mice successfully hindered the communication from bone to liver and rescued the observed elevation in serum cholesterol. Together, our results identify an essential role for bone‐derived SAA3 to regulate liver function and cholesterol homeostasis.

## 2 Results

### 2.1 Genetic Depletion of *Tsc1* in Osteocytes/Osteoblasts is Associated with Elevated Serum Cholesterol Level in Mice

**Figure 1:**
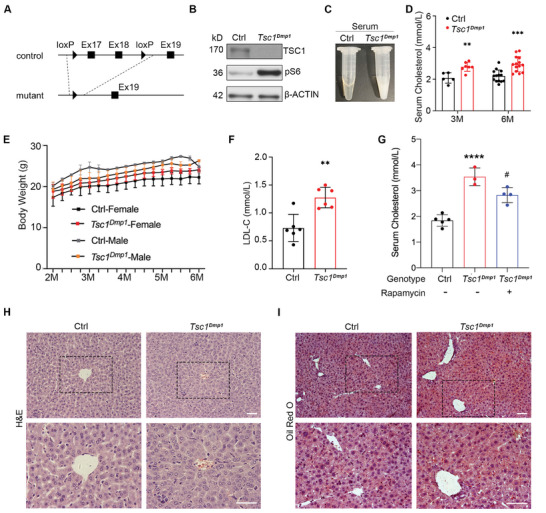
Genetic depletion of *Tsc1* in osteocytes/osteoblasts is associated with elevated serum cholesterol level in mice. A) Cre‐mediated recombination eliminates exons 17–18 of *Tsc1* gene. B) Western blot of TSC1 and pS6 expression levels in cortical bone samples from *Tsc1^Dmp1^* mice and control littermates. C) Appearance of serum samples collected from *Tsc1^Dmp1^* mice and control littermates at 6 months of age. D) Serum cholesterol concentrations in *Tsc1^Dmp1^* mice and control littermates at 3 or 6 months of age (n = 5–14, Ctrl (3 m): 3 females, 2 males; *Tsc1^Dmp1^* (3 m): 4 females, 3 males; Ctrl (6 m): 6 females, 8 males; *Tsc1^Dmp1^* (6 m): 9 females, 5 males). E) Body weight curves of *Tsc1^Dmp1^* mice and control littermates from 2 to 6 months of age (n = 6, 4 females, 2 males). F) Serum LDL‐C concentrations in *Tsc1^Dmp1^* mice and control littermates at 6 months of age (n = 6, 4 females, 2 males). G) Serum cholesterol concentrations in *Tsc1^Dmp1^* mice and control littermates treated with or without rapamycin (2 mg kg^−1^ of body weight/day for 2 weeks) at 9 months of age (n = 3–5, Ctrl: 4 females, 1 male; *Tsc1^Dmp1^* without rapamycin: 2 females, 1 male; *Tsc1^Dmp1^* with rapamycin: 3 females, 1 male). H) Representative H&E staining images of paraffin sections of liver from 6‐month‐old *Tsc1^Dmp1^* mice and control littermates. I) Representative Oil red O staining images in frozen liver sections of 6‐month‐old *Tsc1^Dmp1^* mice and control littermates. Scale bars, 100 µm; data represent mean ± SD; each symbol represents one animal. ^*^ *p* < 0.05, ^**^ *p* < 0.01, ^***^ *p* < 0.001 compared to Ctrl; # p < 0.05 compared to *Tsc1^Dmp1^*, by unpaired t test (D‐F) or two‐way ANOVA (G).

The TSC1‐mTORC1 signaling axis integrates stimulating inputs from nutrients and growth factors to regulate bone development, homeostasis, and function. In previous work we established *Tsc1^Dmp1^* mice (Figures S1,  S2, and S3A, Supporting Information), where 17th to 18th exons of *Tsc1* gene was knocked out in osteocytes/osteoblasts (**Figure** **1**A). The phosphorylation of ribosomal S6 protein at S235/S236 (pS6) was evaluated to verify the activation of mTORC1 signaling pathway (Figure 1B; Figure S1F, Supporting Information). Interestingly, the serum samples of *Tsc1^Dmp1^* mice appeared white and opaque (Figure 1C; Figure S1H, Supporting Information), serum levels of total cholesterol (34% and 40% increases at 3 and 6 mo respectively, Figure 1D), low‐density lipoprotein cholesterol (LDL‐C) (74% increase at 6 mo, Figure 1F), and triglyceride (200% increase at 6 mo, Figure S1I, Supporting Information) were significantly elevated in *Tsc1^Dmp1^* mice, while body weight (Figure 1E) and high‐density lipoprotein cholesterol (HDL‐C) concentrations (Figure S1J, Supporting Information) retained the same. Indeed, TSC1 has the potential to exert functions that are both dependent on and independent of mTORC1. To investigate whether the observed metabolic phenotype is mediated via the TSC1‐mTORC1 signaling pathway, we administered rapamycin (2 mg kg^−1^ of body weight/day for 2 weeks) to both *Tsc1^Dmp1^* mice and control littermates through intraperitoneal injection to specifically inhibit mTORC1. Evidently, the administration of rapamycin significantly impeded the elevation of serum cholesterol levels in *Tsc1^Dmp1^* mice (Figure 1G). For the most part, males and females had similar phenotypic responses to genetic depletion of *Tsc1* in osteocytes/osteoblasts and administration of rapamycin.

In addition, as hypercholesterolemia is often accompanied by cardiovascular diseases, for example, atherosclerosis, we isolated whole aortas from 13‐month‐old *Tsc1^Dmp1^* male mice and evaluated the existence of atherosclerotic lesions via Oil Red O staining. As shown by the arterial tree stained with Oil Red O (Figure S1K, Supporting Information, middle) and the longitudinally split stained whole aorta (Figure S1K, Supporting Information, right), the observed small pieces of stained tissues were adventitial fat that remained attached to the aorta, and no atherosclerotic lesions were observed in aortas of either *Tsc1^Dmp1^* mice or control littermates (Figure S1K, Supporting Information). Given the critical role for liver in managing cholesterol production and clearance, the changes in liver anatomy and lipid droplet morphology were next examined. As indicated by H&E and Oil red O staining results, liver sections from control and *Tsc1^Dmp1^* mice share similar morphological characteristics (Figure 1H,I). Thus, we hypothesized that osteocytes/osteoblasts may play an unappreciated role in regulating cholesterol metabolism without altering liver morphology.

### 2.2 Loss of *Tsc1* Induces Expression and Secretion of SAA3 in Osteocytes/Osteoblasts

**Figure 2:**
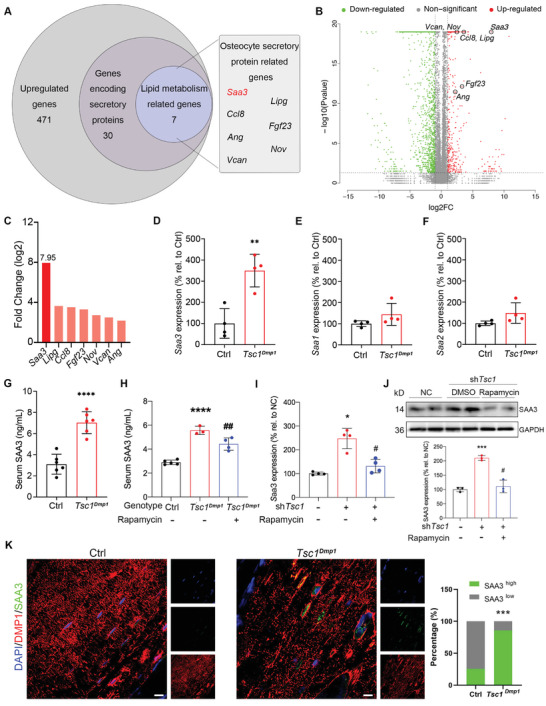
Loss of *Tsc1* induces expression and secretion of SAA3 in osteocytes/osteoblasts. A) Venn diagram showing categories of upregulated genes (FDR < 0.05, RNA‐seq) in cortical bone samples collected from 3‐month‐old *Tsc1^Dmp1^* mice comparing to control littermates. Outlined box on the right shows the 7 genes that are known to be related to lipid metabolism. B) Volcano plot showing differences in gene expression profile between cortical bone samples collected from 3‐month‐old *Tsc1^Dmp1^* mice and control littermates, labeled *Saa3, Lipg, Ccl8, Fgf23, Nov, Vcan, and Ang*. C) Expression levels of the 7 upregulated lipid metabolism related genes identified in transcriptome analysis. D,E) Relative mRNA expression levels (qRT‐PCR) of *Saa1* (E), *Saa2* F), and *Saa3* (D) in mouse cortical bones collected from 3‐month‐old *Tsc1^Dmp1^* mice or control littermates (n = 4, 3 females, 1 male). G) Quantification of serum SAA3 concentration (ELISA) in 3‐month‐old *Tsc1^Dmp1^* mice and control littermates (n = 6, 4 females, 2 male). H) Quantification of serum SAA3 concentration (ELISA) in 9‐month‐old *Tsc1^Dmp1^* mice and control littermates treated with or without rapamycin (2 mg kg^−1^ of body weight/day for 2 weeks) (n = 3–5, Ctrl: 4 females, 1 male; *Tsc1^Dmp1^* without rapamycin: 2 females, 1 male; *Tsc1^Dmp1^* with rapamycin: 3 females, 1 male). I,J) *Saa3*/SAA3 mRNA (I) or protein (J) levels in indicated mouse osteocyte Mlo‐Y4 cell group (n = 3–4 per group). K) Representative immunofluorescence staining images of DMP1/SAA3 co‐localization in mouse femur paraffin sections and quantification of cells with different SAA3 expression levels (n = 3, 2 females, 1 male). Left panel insets show individual staining images. Scale bars, 50 µm; data represent mean ± SD; each symbol represents one animal or one well. ^*^ *p* < 0.05, ^**^ *p* < 0.01, ^***^ *p* < 0.001 compared to Ctrl; # p < 0.05, ## p < 0.01 compared to *Tsc1^Dmp1^* or *shTsc1*, by unpaired t test (D‐G, K) or two‐way ANOVA (H‐J).

In light of the data supporting a potential role for osteocytes/osteoblasts in the regulation of hepatic metabolism of cholesterol, RNA sequencing was performed using cortical bones to explore possible osteocytes/osteoblasts‐derived factors. Differential expression analysis of RNA transcripts revealed significant differences between samples from *Tsc1^Dmp1^* and control littermates. A set of 471 genes was identified to be upregulated in cortical bone of *Tsc1^Dmp1^* mice (Table S1, Supporting Information), among which 30 genes were known to encode secretory proteins (**Figure** **2**A). Molecular function pathway enrichment analysis of the 30 upregulated secretory protein‐related genes revealed a set of 7 genes that are related to lipid metabolism (Figures 2A,B). Among these, *Saa3* was the most upregulated transcript in *Tsc1^Dmp1^* group (2.99‐fold increase vs control, Figure 2C). The mouse SAA family contains four conservative genes: *Saa1‐4*, among which functional products of *Saa1‐3* are known to play roles in cholesterol metabolism,^[^ ^19^ ^]^ and *Saa4* encodes for a constitutively expressed protein.^[^ ^20^ ^]^ In cortical bone samples, only *Saa3* was upregulated by *Tsc1* ablation (Figure 2D–F). Consistent with this, a significant increase in the proportion of SAA3‐expressing osteocytes/osteoblasts (Figure 2K) and a 2.27‐fold elevation in serum SAA3 concentration (Figure 2G), which were evaluated using immunofluorescence staining or enzyme‐linked immunosorbent assay (ELISA) analysis respectively, confirmed the production and secretion of SAA3 at cortical bone after the genetic depletion of *Tsc1* in osteocytes/osteoblasts. In addition, the inhibition of mTORC1 signaling pathway via rapamycin administration (2 mg kg^−1^ of body weight/day for 2 weeks for mice, 100 nm for 24 h for Mlo‐Y4 cells) resulted in the attenuation of increased serum SAA3 levels in *Tsc1^Dmp1^* mice (Figure 2H), as well as the overexpression of SAA3 in *Tsc1*‐knockdown Mlo‐Y4 cells (Figure 2I–J; Figure S3C,D, Supporting Information), implying that the overproduction of SAA3 is indeed mediated through the TSC1‐mTORC1 pathway.

Notably, as adipose tissue and the liver are widely viewed as the primary sources of SAA3 in response to stimuli,^[^ ^21^ ^]^ and *Dmp1‐Cre* is known to have unintended targeting,^[^ ^22^ ^]^ experiments were conducted to examine the involvement of adipose tissue and the liver in SAA3 production. First, *mTmG^Dmp1^* mice were constructed to detect *Dmp1‐Cre* activity in major organs including the liver (Figure S2B, Supporting Information). In consistent with a previous study using *Ai9* reporter line,^[^ ^22b^ ^]^ no green (*Dmp1^Cre^/mTmG^fl/fl^* mice, Figure S2B, Supporting Information) or red (*Dmp1^Cre^/Ai9* mice) ^[^ ^22b^ ^]^ fluorescence was detected in the liver. Thus, *Dmp1^Cre^*‐mediated ablation of *Tsc1* should not directly affect molecular events within the liver. In addition, we performed western blot analyses using visceral adipose tissues from *Tsc1^Dmp1^* mice and control littermates. No significant difference was observed between these two groups (Figure S2C, Supporting Information). Together, our results eliminated the involvement of the known sources of SAA3 in response to stimuli – adipose tissue and the liver.

### 2.3 Exogenous Recombinant SAA3 Elevates Serum Cholesterol Level In Vivo

**Figure 3:**
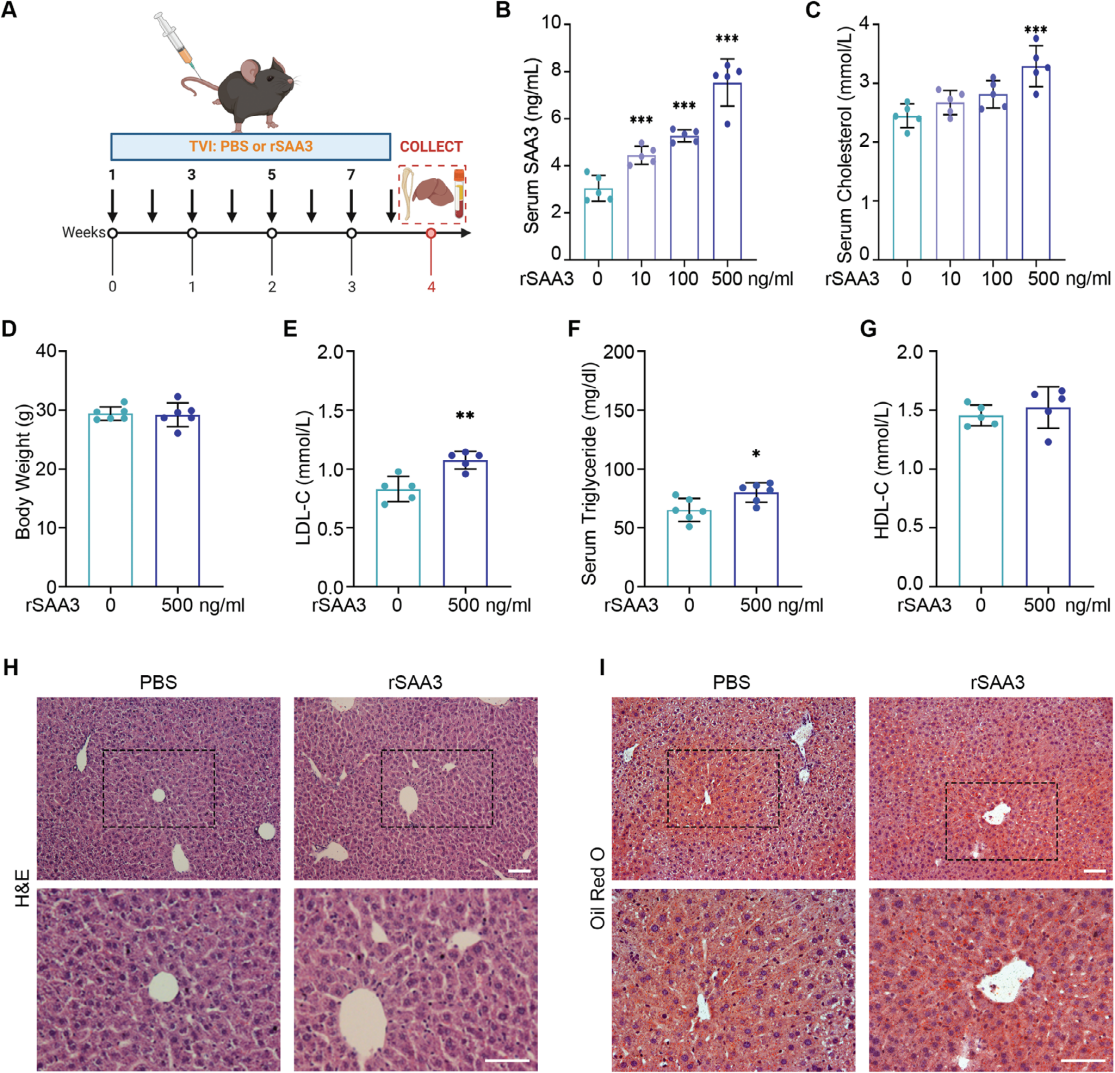
Exogenous recombinant SAA3 elevates serum cholesterol level in vivo. A) Schematic illustrating the exogenous rSAA3 (tail vein injection) experimental design and downstream sample collection. B) Quantification of serum SAA3 concentration (ELISA) in C57BL/6 mice 3 h after the last tail vein injection of different doses of rSAA3 protein (n = 5, 5 males). C) Quantification of serum cholesterol levels of C57BL/6 mice after tail vein injection of different doses of rSAA3 protein (n = 5, 5 males). D) Body weight of mice injected with 500 ng mL^−1^ of rSAA3 protein or PBS (n = 6, 6 males). E–G) Quantification of serum triglyceride (F), LDL‐C (E) and HDL‐C (G) levels in mice injected with 500 ng mL^−1^ of rSAA3 protein or PBS (n = 5–6, 5–6 males). H) Representative H&E staining images of paraffin sections of liver after tail vein injection of 500 ng mL^−1^ of rSAA3. I) Representative Oil red O staining images of frozen liver sections after tail vein injection of 500 ng mL^−1^ of rSAA3. Bottom panel insets show magnification. Scale bars, 100 µm; data represent mean ± SD; each symbol represents one animal. ^*^ *p* < 0.05, ^**^ *p* < 0.01, ^***^ *p* < 0.001, by one‐way ANOVA (B‐C) or unpaired t test (D‐G).

SAA3 impacts on cholesterol homeostasis were further confirmed and explored, particularly with respect to phonotypes observed in *Tsc1^Dmp1^* mice. Due to the short half‐life of SAA proteins (30–120 min),^[^ ^23^ ^]^ relatively high concentrations (10, 100, and 500 ng mL^−1^) of recombinant SAA3 (rSAA3) were used and periodic injections were performed. Briefly, C57BL/6 mice were injected with indicated dosage of rSAA3 twice a week for 4 weeks through tail vein, and samples were collected after the 4‐week treatment (**Figure** **3**A; Figure S3B, Supporting Information). Male C57BL/6 mice were used to avoid the effects of estrogen. At the end of this 4‐week treatment, samples were collected 4 h after the last injection. As expected, ELISA revealed elevating SAA3 concentrations in serum with the increase of injection dosage (Figure 3B). In this setting, elevations in serum cholesterol level (Figure 3C), as well as LDL‐C concentration (Figure 3E), occurred with exogenous rSAA3 tail vein injection in a dose‐dependent manner, while HDL‐C concentration (Figure 3G) and body weight (Figure 3D) remained unchanged, suggesting an essential role for SAA3 in disturbing cholesterol homeostasis and consistent with that in *Tsc1^Dmp1^* mice. Similarly, exogenous rSAA3 did not alter liver anatomy or lipid droplet morphology of the treated mice (Figure 3H,I), supporting the role of SAA3 in the observed hypercholesterolaemia in *Tsc1^Dmp1^* mice.

### 2.4 *Tsc1^Dmp1^* and rSAA3‐Treated Mice Exhibit Reduction in Hepatic CYP7A1

**Figure 4:**
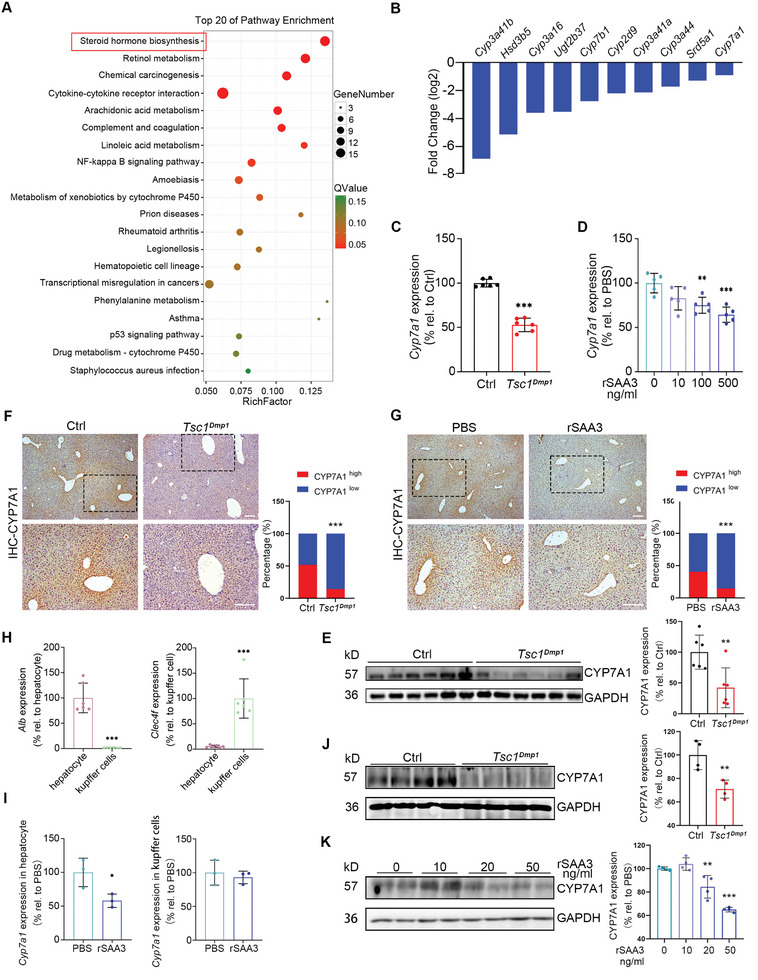
*Tsc1^Dmp1^* and rSAA3‐treated mice exhibit reduction in hepatic CYP7A1. A) Transcriptional analysis of liver samples collected from 3‐month‐old *Tsc1^Dmp1^* mice or control littermates. Diagram shows the top 20 KEGG pathways enriched. The size and color of bubbles represent the number and degree of enrichment of differentially expressed mRNA enriched in the pathway. B) Expression levels of the top 10 downregulated lipid metabolism related genes identified in transcriptome analysis. C) Relative mRNA expression levels (qRT‐PCR) of *Cyp7a1* in liver samples collected from 6‐month‐old *Tsc1^Dmp1^* mice or control littermates (n = 6, 4 females, 2 males). D) Relative mRNA expression levels (qRT‐PCR) of *Cyp7a1* in liver samples collected from mice treated with 500 ng mL^−1^ of rSAA3 or PBS (n = 5, 5 males). E) Western blot analysis of CYP7A1 in liver samples collected from 6‐month‐old *Tsc1^Dmp1^* mice or control littermates (n = 6, 4 females, 2 males). F) Representative immunohistochemical staining images of CYP7A1 in paraffin sections of liver samples collected from 6‐month‐old *Tsc1^Dmp1^* mice or control littermates and quantification of cells with different CYP7A1 expression levels (n = 4, 3 females, 1 male). G) Representative immunohistochemical staining of CYP7A1 in paraffin sections of liver samples collected from mice treated with 500 ng mL^−1^ of rSAA3 or PBS (n = 4, 4 males). H) Relative mRNA expression levels (qRT‐PCR) of *Alb* and *Clec4f* in hepatocytes and Kupffer cells (n = 6, 6 males). I) Relative mRNA expression levels (qRT‐PCR) of *Cyp7a1* in hepatocytes and Kupffer cells (n = 3, 3 males). J) Western blot analysis of CYP7A1 in Hepa1‐6 cells treated with serum samples collected from 6‐month‐old *Tsc1^Dmp1^* mice or control littermates (n = 4, 3 females, 1 male). K) Western blot analysis of CYP7A1 in Hepa1‐6 cells treated with 50 ng mL^−1^ of rSAA3 (n = 4). Scale bars, 200 µm; data represent mean ± SD; each symbol represents one animal. ^*^ *p* < 0.05, ^**^ *p* < 0.01, ^***^ *p* < 0.001, by one‐way ANOVA (D, K) or unpaired t test (C, E‐J).

Liver is the major organ involved in endogenous biosynthesis and clearance of cholesterol. To investigate mechanisms of the observed hypercholesterolaemia in *Tsc1^Dmp1^* mice, RNA sequencing was performed using whole liver tissues collected from *Tsc1^Dmp1^* mice and control littermates. Kyoto Encyclopedia of Genes and Genomes (KEGG) analysis of the 510 differentially expressed genes in *Tsc1^Dmp1^* mice versus control littermates revealed significant differences in the expression of genes related to the biosynthesis of steroid hormones and metabolism of xenobiotics by cytochrome P450 (**Figure** **4**A). Notably, among the downregulated genes (Figure 4B), *Cyp7a1* and *Cyp7b1*, especially *Cyp7a1*, are well‐known for their essential roles in cholesterol catabolism. CYP7A1 is the initial and rate‐limiting enzyme in the bile acid biosynthesis process, converting and thus clearing serum cholesterol. In comparison with control littermates, *Tsc1^Dmp1^* mice exhibited reductions in both mRNA and protein levels of hepatic CYP7A1 (Figures 4C,E,F), likely partially explaining the observed accumulation of serum cholesterol in *Tsc1^Dmp1^* mice. In consistent with *Tsc1^Dmp1^* mice, rSAA3‐treated mice also demonstrated reduction in hepatic CYP7A1 (Figures 4D,G), further supporting the role of SAA3 in bone‐derived regulation on hepatic cholesterol metabolism.

To dissect the hepatic cell type that is responsible for the reduction in CYP7A1, Percoll density gradient centrifugation was performed to separate hepatocytes and Kupffer cells,^[^ ^24^ ^]^ and the mRNA levels of *albumin* (*Alb*) and *C‐type lectin receptor* (*Clec4f*) were measured to evaluate the successfulness of separation (Figure 4H). Comparing hepatocytes and Kupffer cells from control and rSAA3‐treated mice, data demonstrated significantly different *Cyp7a1* expression profile in hepatocytes (Figure 4I). Consistent with this, in Hepa1‐6 cells, a murine hepatoma cell line, treatment with rSAA3 or serum from *Tsc1^Dmp1^* mice successfully reduced the protein level of CPY7A1 (Figure 4J,K), further supporting the involvement of hepatocytes in the observed bone‐liver crosstalk. Together, these results indicate that genetic depletion of TSC1 in osteocytes/osteoblasts stimulates their production and secretion of SAA3, causing a reduction of CYP7A1 in hepatocytes.

### 2.5 SAA3 Binds to TLR4 on Hepatocytes and Phosphorylates c‐Jun to Downregulate CYP7A1

**Figure 5:**
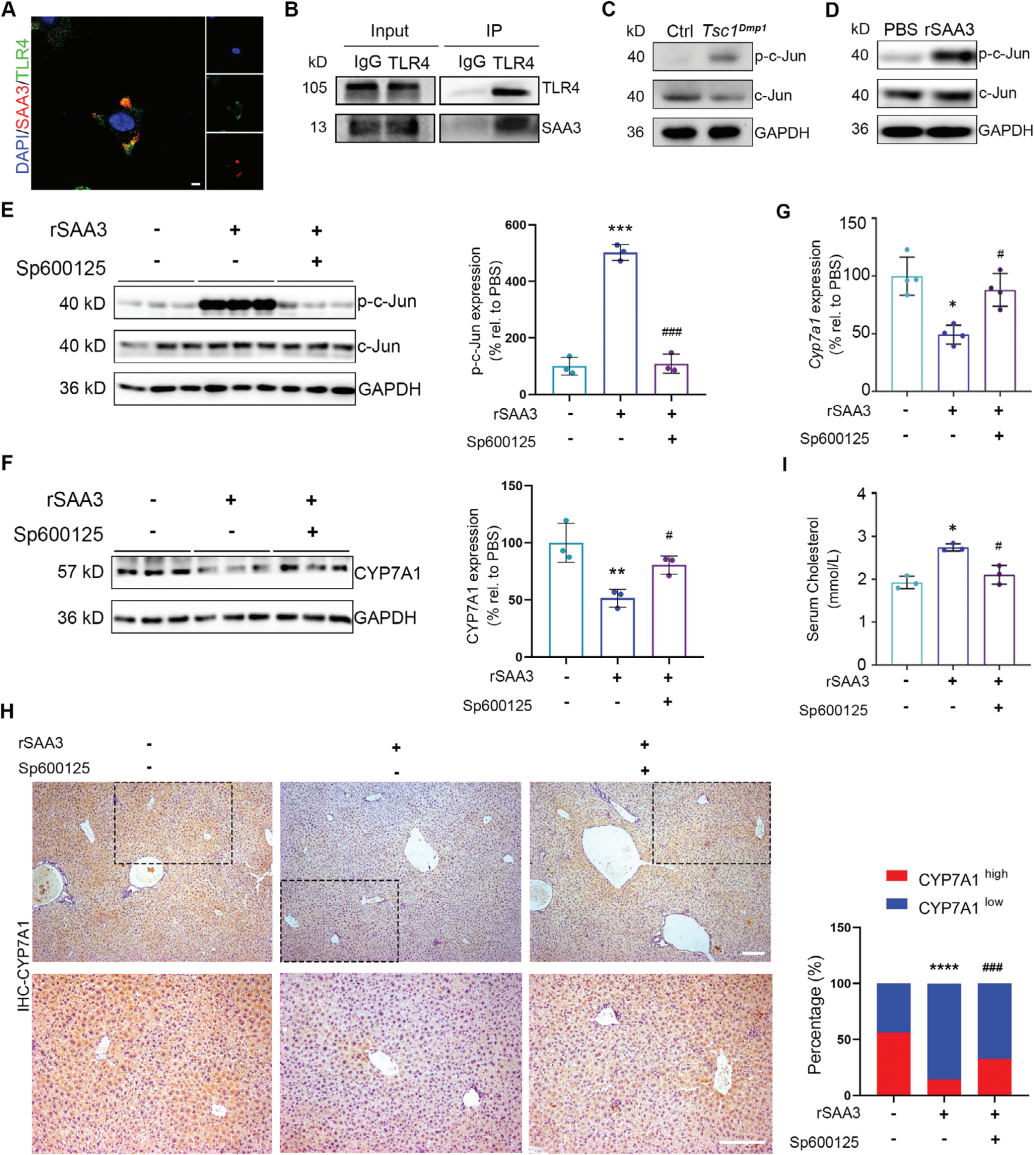
SAA3 binds to TLR4 on hepatocytes and phosphorylates c‐Jun to downregulate CYP7A1. A) Representative immunofluorescence staining images of TLR4/SAA3 co‐localization in Hepa1‐6 cells treated with 50 ng mL^−1^ of rSAA3. Left panel insets show individual staining images. B) Representative result of western blot analysis following immunoprecipitation assay detecting the binding effect of TLR4 and SAA3 in Hepa1‐6 cells treated with 50 ng mL^−1^ of rSAA3. C) Representative result of western blot analysis of p‐c‐Jun in liver samples collected from 6‐month‐old *Tsc1^Dmp1^* mice or control littermates. D) Representative result of western blot analysis of p‐c‐Jun in Hepa1‐6 cells treated with 50 ng mL^−1^ of rSAA3 or PBS. E) Western blot analysis of c‐Jun phosphorylation in Hepa1‐6 cells treated with 50 µm of Sp600125 in the presence or absence of SAA3 stimulation (n = 3). Right panel shows quantification. F) Western blot analysis of CYP7A1 in liver samples collected from mice treated with 5 mg kg^−1^ of Sp600125 in the presence or absence of SAA3 treatment (n = 3, 3 males). Right panel shows quantification. G) Relative mRNA expression levels (qRT‐PCR) of *Cyp7a1* in Hepa1‐6 cells treated with 50 µm of Sp600125 in the presence or absence of SAA3 stimulation (n = 4). H) Representative immunohistochemical staining images of CYP7A1 in paraffin sections of liver samples collected from mice treated with 5 mg kg^−1^ of Sp600125 in the presence or absence of SAA3 treatment. Right panel shows quantification of cells with different CYP7A1 expression levels (n = 3, 3 males). I) Serum cholesterol concentrations in C57BL/6 mice treated with 5 mg kg^−1^ of Sp600125 in the presence or absence of SAA3 treatment (n = 3, 3 males). Scale bars, 50 µm (A); Scale bars, 200 µm (H); data represent mean ± SD; each symbol represents one animal. ^*^ *p* < 0.05, ^**^ *p* < 0.01, ^***^ *p* < 0.001, ^*****^ *p* < 0.0001 compared to rSAA3^−^/Sp600125^−^, #*p* < 0.05, ### p < 0.001 compared to rSAA3^+^/Sp600125^−^, by two‐way ANOVA (E‐I).

To identify the SAA3‐binding protein with roles in the observed reduction in hepatic CYP7A1, predictions on gene network and protein–protein association were constructed using both GeneMANIA and STRING databases (Figure S3C, Supporting Information). The analyses identified four potential SAA3‐interacting proteins, among which TLR4 was validated by colocalization in immunofluorescence staining and immunoprecipitation (**Figure** **5**A,B). The involvement of TLR4 was further confirmed by evaluating the phosphorylation state of c‐Jun, a well‐characterized downstream target of TLR4. Western blotting analysis using whole liver tissue from *Tsc1^Dmp1^* mice and littermates revealed elevation in phosphorylated c‐Jun (Figure 5C). Consistent with this, rSAA3‐treated Hepa1‐6 cells also had an increase in p‐c‐Jun (Figure 5D), suggesting that TLR4‐c‐Jun is likely downstream of SAA3 binding.

The involvement of TLR4‐c‐Jun in *Tsc1^Dmp1^*‐induced hypercholesterolaemia was next examined. In vitro experiments using Hepa1‐6 cells demonstrated that by blocking c‐Jun N‐terminal kinase (JNK) with Sp600125 (Figure 5E; Figure S3D, Supporting Information),^[^ ^25^ ^]^ the rSAA3‐indcued reduction in CYP7A1 was prevented (Figure 5F). Consistent with this, hepatic CYP7A1 level (Figure 5G,H) as well as serum cholesterol level were retained in mice treated with both rSAA3 and Sp600125 (Figure 4I), confirming the role of SAA3‐TLR4‐c‐Jun in regulating cholesterol clearance.

### 2.6 TSC1 Deficiency‐Enabled Crosstalk from Bone to Liver Requires SAA3

**Figure 6:**
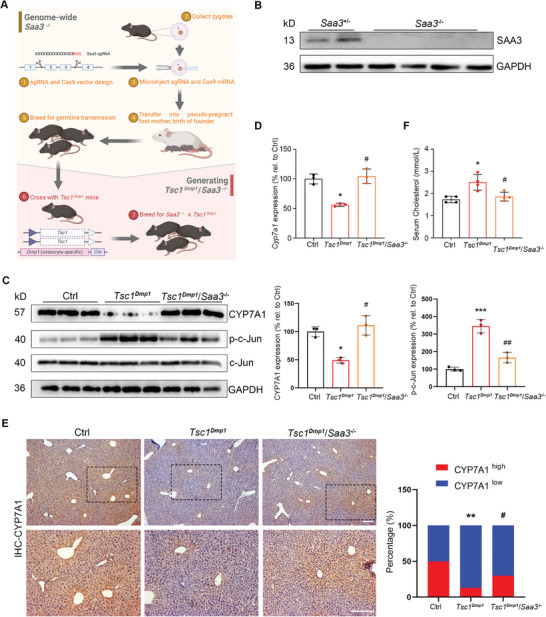
TSC1 deficiency‐enabled crosstalk from bone to liver requires SAA3. A) Schematic illustrating the reproductive strategy in generating *Tsc1^Dmp1^/Saa3* ^−^ *^/^* ^−^ mice. B) Knockout validation was monitored with the SAA3 levels by western blot analysis. C) Western blot analysis of CYP7A1, p‐c‐Jun, and c‐Jun in liver samples collected from 6‐month‐old *Tsc1^Dmp1^/Saa3* ^−^ *^/^* ^−^ mice or indicated control strain (n = 3, 2 females, 1 male). D) Relative mRNA expression levels (qRT‐PCR) of *Cyp7a1* in liver samples collected from 6‐month‐old *Tsc1^Dmp1^/Saa3* ^−^ *^/^* ^−^ mice or indicated control strain (n = 3, 2 females, 1 male). E) Representative immunohistochemical staining images of CYP7A1 in paraffin sections of liver samples collected from 6‐month‐old *Tsc1^Dmp1^/Saa3* ^−^ *^/^* ^−^ mice or indicated control strain. Right panel shows quantification of cells with different CYP7A1 expression levels (n = 3, 2 females, 1 male). F) Serum cholesterol levels in 6‐month‐old *Tsc1^Dmp1^/Saa3* ^−^ *^/^* ^−^ mice or indicated control strain (n = 4, 2 females, 2 males). Bottom panel insets show magnification. Scale bars, 200 µm; data represent mean ± SD; each symbol represents one animal. ^*^ *p* < 0.05, ^**^ *p* < 0.01, ^***^ *p* < 0.001 compared to Ctrl, # p < 0.05, ## p < 0.01 compared to *Tsc1^Dmp1^*, by two‐way ANOVA (D‐F).

Given the functional experiments showing that bone‐derived SAA3 can alter hepatic cholesterol metabolism, we examined the necessity of SAA3 by generating *Saa3* whole‐body knockout mice with CRISPR‐Cas9 method and crossing with *Dmp1^Cre^/Tsc1^fl/fl^* (*Tsc1^Dmp1^*) mice (**Figure** **6**A). The selected offspring for further investigation (termed *Tsc1^Dmp1^/Saa3* ^−^ *^/^* ^−^) have whole‐body *Saa3* depleted and *Tsc1* knocked out in osteocytes/osteoblasts (Figure 6B). As expected, *Tsc1^Dmp1^/Saa3* ^−^ *^/^* ^−^ mice displayed normal levels of hepatic p‐c‐Jun/c‐Jun, hepatic CYP7A1, and serum cholesterol (Figure 6C–F) compared with control littermates. There was no difference observed between male and female *Tsc1^Dmp1^/Saa3* ^−^ *^/^* ^−^ mice. These data suggest that the observed crosstalk from bone to liver in *Tsc1^Dmp1^* mice requires SAA3.

## 3 Discussion

Here, using two genetically engineered mouse models, we show that osteocytes/osteoblasts produce and secrete SAA3 due to the loss of TSC1 and reduces CYP7A1 expression in hepatocytes through TLR4‐c‐Jun signaling axis, thus impeding hepatic cholesterol catabolism and resulting in hypercholesterolaemia. In this setting, SAA3‐mediated bone‐liver crosstalk acts as an essential regulatory mechanism to control cholesterol homeostasis. These data expand our understanding of the crosstalk from bone to distant organs and provide a rationale to search for additional bone‐secreting proteins affecting liver functions to dissect the physiologic, cellular, and molecular pathology of the cooccurrence of bone and liver disorders.

Bone is generally stereotyped as the rigid, supportive, and protective organ for the body. Nonetheless, clinical research has found that bone diseases are associated with a variety of disorders, for example, osteoporosis and dementia are associated with increased incidence rate of Alzheimer's disease. In this regard, studies have been conducted to reveal the function and importance of bone as a secretory organ. It was first recognized that bone acts as an endocrine organ to regulate systemic mineral metabolism. Besides, as an endocrine organ, prior work supports a role for bone in glucose metabolism and energy expenditure. Osteocytes synthesize osteocalcin (OC), which is decarboxylated to form undercarboxylated osteocalcin (GluOC) during bone resorption. GluOC promotes *β*‐cell proliferation, stimulates insulin synthesis and secretion, increases insulin sensitivity in adipose tissue, muscle, and liver, and leads to increased energy expenditure.^[^ ^26^ ^]^ The present work shows that osteocytes/osteoblasts synthesize and secrete SAA3 due to the disruption of TSC1‐mTORC1 signaling axis, and thus regulates hepatic clearance of cholesterol, adding to the known functions of bone‐derived factors in regulating physiological homeostasis to highlight the role of crosstalk from bone to distant organs. Besides, substantial changes in serum triglyceride levels were also observed in these *Tsc1^Dmp1^* mice. Understanding the underlying mechanisms should provide new insights into the significance of bone‐derived factors.

mTORC1 signaling axis links external stimuli to bone development, growth, and function. Its critical roles in regulating bone cell perception and nutrient supply have been well established. For example, mechanical stress and muscle contraction induced by exercise stimulation activate mTORC1 to promote bone cell proliferation, bone matrix synthesis, and bone remodeling;^[^ ^27^ ^]^ stimuli from nutrients, such as amino acids and glucose, activate mTORC1 to promote the proliferation and growth of bone cells.^[^ ^28^ ^]^ However, the importance of mTORC1 in the regulation of bone‐derived crosstalk to distant organs have yet to be dissected. Our prior work indicated that mice with constitutive activation of mTORC1 signaling in osteocytes exhibited a dramatic increase in IL‐19 production, and in turn stimulated granulopoiesis and neutrophil formation.^[^ ^5c^ ^]^ On the other hand, the present work shows that dysregulation of TSC1‐mTORC1 in osteocytes/osteoblasts induces the production and secretion of SAA3 to regulate hepatic cholesterol catabolism, supporting the multifunctional property of mTORC1 signaling axis. While our molecular analyses were largely focused on the downstream signaling pathway of SAA3 and the observed hypercholesterolaemic phenotype, we have not yet defined details of the upregulation mechanisms of SAA3 by TSC1 ablation in osteocytes/osteoblasts. Notably, mTORC1 is widely viewed as a key modulator for the activation of nuclear factor‐κB (NF‐κB) signaling in varies of tissues,^[^ ^5^, ^29^ ^]^ and we have observed strongly activated NF‐κB activity in TSC1‐null osteocytes/osteoblasts in our previous study using *Tsc1^Dmp1^* mice.^[^ ^5c^ ^]^ In combination with the fact that transcription of SAA3 was shown to be synergistically induced by NF‐κB binding to SAA3 enhancer factor,^[^ ^30^ ^]^ this suggests that TSC1 ablation may work through mTORC1‐NF‐κB mechanisms to modulate SAA3 production in osteocytes/osteoblasts. Understanding the precise mechanisms by which SAA3 levels are elevated in TSC1‐null osteocytes/osteoblasts should be a priority for future studies.

Both in vivo and in vitro data here identified the importance of SAA3‐TLR4‐CYP7A1 in regulating hepatic cholesterol catabolism. SAA is a family of apolipoproteins that share high levels of sequence homology. Clinically, epidemiological data have linked SAA with cardiovascular diseases and related mortality.^[^ ^31^ ^]^ Animal studies have further identified the causal relationship between SAA and atherosclerosis,^[^ ^32^ ^]^ potentially through pro‐inflammatory and pro‐atherogenic activities.^[^ ^19^, ^33^ ^]^ Moreover, SAAs are considered to be biomarkers for tumor progression and reduced survival in many human cancers,^[^ ^34^ ^]^ and SAA1/SAA3 have been proposed to play a key role in mediating the protumorigenic properties of cancer‐associated fibroblasts in pancreatic tumors.^[^ ^35^ ^]^ Findings here implicating the regulatory function of bone‐derived SAA3 on hepatic cholesterol clearance uncover a mechanism of SAA3 action as a biomolecular cue in regulating cholesterol homeostasis in mice. Notably, our molecular analysis was largely limited to the *Tsc1^Dmp1^* mice; while this is the primary focus of our present work, exploring the effects of rSAA3 on liver function could provide valuable confirmation. We have not yet identified the specific cellular or molecular mechanisms of rSAA3 supplementation responsible for the elevatory effects on serum cholesterol levels, and this is a critical area for future research. In humans, the genome only contains three SAA family genes that encode functional proteins: *Saa1*, *Saa2*, and *Saa4*,^[^ ^36^ ^]^ among which *Saa4* is constitutively expressed.^[^ ^20^ ^]^ It also contains *Saa3* as a pseudogene,^[^ ^37^ ^]^ which has very similar sequences as *Saa1* and *Saa2*.^[^ ^19^, ^38^ ^]^ Thus, examining the expression levels of TSC1‐mTORC1 axis in bone and protein levels of SAA1/SAA2 in serum of hypercholesterolaemic patients may provide new insight into both biological mechanisms and translatability. It will be of interest to determine if the skeletal secretion of SAA3 that is seen in this specific genetical disturbance setting, affects hepatic cholesterol clearance in the context of bone‐related diseases.

## 4 Experimental Section

### Animals

Animal experiments were approved by the Ethical Committee for Animal Research of Southern Medical University and conducted according to the state guidelines from the Ministry of Science and Technology of China. C57BL/6 mice were purchased from the Laboratory Animal Centre of Southern Medical University (Guangzhou, China). *Tsc1^fl/fl^* and *Dmp1‐Cre* mice (*Dmp1^Cre^*, IMSR_JAX:023047) were purchased from The Jackson Laboratory (Bar Harbor, ME). To generate osteocyte/osteoblast‐specific *Tsc1* deletion mice, *Tsc1^fl/fl^* mice (IMSR_JAX:005680) were crossed with *Dmp1^Cre^* mice. *Tsc1^fl/fl^* mice in the same litter served as control.

The generation of *Saa3* ^−^ *^/^* ^−^ mice was initiated using CRISPR Design Website (http://www.e‐crisp.org/E‐CRISP/) to design knockout targets for the second exon and the third exon of mouse *Saa3* gene. The designed targets were as follows: sgRNA target 1‐CTTCATCCTGCTATAGGGCC and sgRNA target 2–GTCATCAGGTAACACGGGTC. SgRNA and Cas9 mRNA were synthesized in vitro and stored at −80 °C for further use. After superovulation and fertilization, 100 ng µL^−1^ Cas9 mRNA and 50 ng µL^−1^ sgRNA (Saa3‐sgRNA1 and Saa3‐sgRNA2) were injected into C57BL/6J mouse 1‐cell zygote and cultured in K^+^ Simplex Optimised Medium (KSOM) embryo medium at 37 °C. The cultured embryos were transplanted into the fallopian ampullae of pseudopectic ICR female mice and then kept for delivery after the wound was sutured. The tail was clipped 21 days after birth to identify the genotypes of mice, and the same mutant mice were routinely bred in cage until *Saa3* knockout strain was screened. PCR, western blotting was used to verify the knockout effect.

To generate *Tsc1^Dmp1^/Saa3* ^−^ *^/^* ^−^ double knockout mice, *Saa3* ^−^ *^/^* ^−^ mice were crossed with *Tsc1^Dmp1^* mice. *Tsc1^fl/fl^* mice in the same litter served as control.

To specifically disturb TSC1‐mTORC1 signaling pathway, rapamycin (Cat#HY‐10219, MedChemExpress, 2 mg kg^−1^ of body weight/day) was administered to *Tsc1^Dmp1^* mice and control littermates for 2 weeks by intraperitoneal injection before sacrifice.

Sex‐matched littermate mice 12 or 24 weeks of age (half male and half female) were used for experiments unless otherwise noted. All animals were provided with a standard diet and housed in pathogen‐free cages at constant temperature and humidity. The circadian rhythm was maintained at 12 h. The Southern Medical University Animal Care and Use Committee approved all procedures involving mice. All animal procedures involving animals and their care were conducted in accordance with the guidelines of Animal Use and Care of the National Institutes of Health.

### Cell Culture

The murine hepatic cell line, Hepa1‐6 cells were cultured in culture medium: Dulbecco's modified Eagle's medium (DMEM) with 10% fetal bovine serum (FBS), 100 units mL^−1^ penicillin, and 100 µg mL^−1^ streptomycin. Cells were grown at 37 °C in a 5% CO_2_ humid atmosphere. For the recombinant SAA3 treatment experiment, Hepa1‐6 cells were treated with corresponding concentrations of recombinant SAA3 (SAB, 10–50 ng mL^−1^) or PBS (as control) for 6 h, when the cell density reached 70–80%. For the c‐Jun/JNK inhibitor treatment experiment, Hepa1‐6 cells were added with the corresponding concentration of recombinant SAA3 protein and SP600125 (Selleck, 50 µm) or PBS (as control) in the medium at the density of 70–80%. The cells were collected after 6 h of treatment.

The mouse osteocyte cell line, Mlo‐Y4 cells were cultured in culture medium: DMEM with 10% FBS, 100 units per mL penicillin, and 100 µg mL^−1^ streptomycin. Cells were grown at 37 °C in a 5% CO_2_ humid atmosphere. Upon lentivirus transfection, the culture medium was switched to infection medium: DMEM with 5% FBS, 100 units per mL penicillin, and 100 µg mL^−1^ streptomycin. Cells seeded in 12‐well were transfected with lentivirus‐*shNC* or lentivirus‐*shTsc1* at MOI of 50 for 48 h. After 24 h of transfection, cells were treated with or without 100 nm rapamycin for the next 24 h. The lentivirus for short hairpin (shRNA)‐mediated knockdown of TSC1 (*shTsc1*; 5′‐GACACACAGAATAGCTATG‐3′) and non‐silence control lentivirus (*shNC*; 5′‐TTCTCCGAACGTGTCACGT‐3′) were purchased from Shanghai GenePharma.

### Isolation of Kupffer Cells and Hepatocytes

Hepatocyte and Kupffer cell separation were performed using Percoll (Solarbio) density gradient centrifugation method. Briefly, the peritoneal cavity was opened, and the intestines were pushed to the right side using a cotton‐tip. The winged needle was then inserted into the inferior vena cava. The vena cava was clamped using a bulldog clamp to secure the needle. The liver swelled and became discolored as the perfusion started. By cutting the hepatic portal vein, the liver discolored rapidly to a creamy color. The perfusion of the liver was continued with ≈15–20 mL of HBSS‐EGTA buffer for ≈10 min. Digestion of the liver was carried out by transferring the tubing to the type 4 collagenase buffer (Sigma) After the completion of perfusion and digestion, the liver was collected and transferred it to a Petri dish containing cold HBSS‐CaCl_2_ buffer. The removal of capsule of Glisson was conducted on ice. Then the hepatic cells were released by gently cutting the liver lobes until all the big clumps were gone. Cells were filtered through a 100 µm cell strainer using 30 mL of cold HBSS‐CaCl_2_ buffer and keep the cells on ice. After the centrifugation for 3 min at a speed of 50 × g at 4 °C, the Kupffer cells were collected in the supernatant, and the hepatocytes were in the pellet.

### Recombinant SAA3 Protein Treatment

3‐month‐old C57BL/6 mice were injected with the corresponding concentration of recombinant SAA3 protein (SAB) through the caudal vein. Compared with the treatment group, the control group was injected with PBS solution. Recombinant SAA3 protein was dissolved in PBS solution and injected twice a week for 4 weeks.

### c‐Jun/JNK Inhibitor Treatment

3‐month‐old C57BL/6 mice were intraperitoneally injected with SP600125 (Selleck) for 15 mg kg^−1^ body weight per mouse started from the recombinant SAA3 protein treatment. Recombinant SAA3 protein and SP600125 was injected twice a week for 4 weeks.

### Protein Extraction and Western Blot

For total protein extraction, liver or cells were lysed in radioimmunoprecipitation assay (RIPA) buffer containing 50 mm Tris‐HCl pH 8, 150 mm NaCl, 1% Triton X‐100, 0.1% sodium deoxycholate, 0.1% SDS, and 1× protease inhibitor cocktail (Roche). Lysates were boiled in 2× SDS sample buffer. Proteins were separated by SDS‐PAGE and transferred to nitrocellulose membranes (Bio‐Rad). The membranes were blocked with 5% non‐fat milk in Tris‐buffered saline with Tween (TBST) at room temperature for 1 h, and then incubated with primary antibodies overnight at 4 °C, rinsed with TBST for three times, and incubated with secondary antibody for 1 h at room temperature. The proteins were visualized with enhanced chemiluminescence.

### Co‐Immunoprecipitation

Treated cells were lysed in cold CHAPS‐containing lysis buffer [0.3% CHAPS, 40 mm HEPES (pH7.4), 150 mm NaCl, 2 mm ethylenediamindium pyrophosphate, 10 mm sodium glycerophosphate, 50 mm NaF, plus protease inhibitors]. Cells were rotated for 20 min at 4 °C, centrifuged at 12 000 rpm for 10 min at 4 °C, and incubated with primary antibodies overnight at 4 °C with continuous rotation. A 50% slurry of protein G Sepharose (Abclonal, 60 µL) was then added and incubated for an additional 1 h. Immunoprecipitated proteins were denatured by addition of 50 µL of SDS loading buffer and boiling for 5 min. Western blotting analysis was used to verify the interaction between SAA3 and TLR4.

### Immunohistochemistry

Bone tissues from mice were fixed in 4% paraformaldehyde for 24 h and decalcified in 5% EDTA. Then, tissues were embedded in paraffin and sectioned into 4 µm‐thick slices. After deparaffinization and rehydration, sections were incubated in citrate buffer (10 mm citric acid, pH 6.0) for 16 h at 60 °C, followed by 5 min in PBS, and treated with 3% hydrogen peroxide for 15 min. After the 1‐h incubation in 5% BSA, tissue sections were incubated with appropriate primary antibodies overnight at 4 °C, and then with the relevant secondary antibody for 1 h at 37 °C. Finally, DAB (ZSGB‐BIO) was used as the substrate to label the target protein and the nuclei were counterstained using hematoxylin. Immunostained sections were imaged on an Axio Scope A1 microscope (Zeiss) and processed using AxioCam HRc3 S/N 2254 – ZEN 2011 software.

### Immunofluorescence

Cells were fixed in 4% paraformaldehyde and stained with the relevant primary antibodies overnight. After washing in PBS for 10 min for three times, cells were stained with fluorescent dye‐conjugated secondary antibodies (Invitrogen). Nuclei were stained with DAPI (Sigma). Tissues were decalcified in 5% EDTA, embedded in paraffin, sectioned into 4 µm‐thick slices, and deparaffinized and dehydrated through a graded ethanol series. Then, sections were incubated in citrate buffer (10 mm citric acid, pH 6.0) for 16 h at 60 °C. After the 1‐h incubation in 5% BSA, sections were incubated with appropriate primary antibodies overnight at 4 °C, and then with the relevant secondary antibody. Nuclei were stained with DAPI (Sigma). Images were obtained using a confocal laser scanning microscope (Olympus FV1000), equipped with a Hamamatsu camera. Red filters (range 575–615 nm) and green filters (range 500–550 nm) were used for Alexa 594 and Alexa 488 staining, respectively. Images were then processed using FV10‐ASW 3.1 software (Olympus).

### H&E Staining

Haematoxylin‐eosin (H&E) staining was performed to examine liver tissue morphology according to standard protocols. In brief, liver tissues were fixed with 4% paraformaldehyde (PFA) overnight. The fixed tissue samples were then embedded in paraffin and sectioned into slices with 4 µm thickness, which were then dehydrated with different concentrations of ethanol and xylol followed by brief washing and staining of cell nuclei with 5% haematoxylin solution (Leagene) for 10 min. After rinsing in distilled water for 5 min, the stained samples were incubated in 0.1% HCl‐ethanol for 30 s. The samples were then counterstained with eosin solution (Leagene) for 2 min. After washing and dehydration, the HE‐stained sections were imaged on an Axio Scope A1 microscope (Zeiss) and processed using AxioCam HRc3 S/N 2254 – ZEN 2011 software.

### Oil Red O Staining

Oil Red O staining was applied to assess lipid droplet formation in liver tissues and aortas. The mouse liver tissues were first fixed in 4% paraformaldehyde and then embedded in paraffin and sectioned into slices with 4‐µm thickness. These sections were then de‐waxed in xylene and rehydrated. After rinsing with PBS, the tissue sections were incubated in Oil Red O reagent for 30 min, followed by haematoxylin counter‐staining for 1 min. After washing and dehydration, the Oil Red O and haematoxylin‐stained sections were mounted Axio Scope A1 microscope (Zeiss) for imaging. To evaluate the existence of atherosclerotic lesions, the aorta from the aortic arch to the abdominal aorta was carefully dissected, placed in a small dish containing PBS, and transferred under a dissecting microscope. Under the dissecting microscope, microscissors and microforceps were used to meticulously remove the fat tissue outside the arterial wall until no obvious fat was observed. The aorta was then transferred to 4% paraformaldehyde and fixed for 30 min, and moved to freshly prepared 60% isopropanol for pre‐rinsing for 10 min. For staining, the aorta was transferred to the working solution of fresh Oil Red O and stained in the dark for 30 min and was then moved to freshly prepared 60% isopropanol for washing for 15 min until most of the nonspecific staining was washed away. Finally, the aorta was placed on a glass slide and photographed under a dissecting microscope.

### qRT‐PCR

Total RNAs from indicated tissues were extracted using TRIzol reagent (Thermo Fisher Scientific) and then reverse‐transcribed into cDNA using HIScript QRT MIX for qPCR (+gDNA wiper) (Vazyme). qPCR was performed using the SYBR‐Green Master PCR Mix (Vazyme). The analyses of all qRT‐PCR results used *Gapdh* as the internal regulator and calculated using 2^−ΔΔCt^ method. Primers used in the present study are listed in key resources table.

### Quantification and Statistical Analysis

Statistical analyses were performed using GraphPad Prim 8.0 software. All data are shown as the mean ± SEM and a p value < 0.05 was considered as significant. For differences between two groups, unpaired t test was used. For analyzing data with three or more groups, one‐way Analysis of Variance (ANOVA) with Tukey's multiple comparison test was used to determine the effects of one independent variable, and two‐way ANOVA with Tukey's multiple comparison test was used to determine the effects of two variables.

### RNA Sequencing

After total RNA was extracted, eukaryotic mRNA was enriched by Oligo(dT) beads, while prokaryotic mRNA was enriched by removing rRNA by Ribo‐ZeroTM Magnetic Kit (Epicentre). Then the enriched mRNA was fragmented into short fragments using fragmentation buffer and reverse‐transcribed into cDNA with random primers. Second‐strand cDNA was synthesized by DNA polymerase I, RNase H, dNTP and buffer. Then the cDNA fragments were purified with QiaQuick PCR extraction kit, end repaired, poly(A) added, and ligated to Illumina sequencing adapters. The ligation products were size selected by agarose gel electrophoresis, PCR amplified, and sequenced using Illumina HiSeqTM 2500 by Gene Denovo Biotechnology Co. (Guangzhou, China).

### SAA3 ELISA

Blood was collected using an RNA‐free EP tube and centrifuged at room temperature at 3000 RPM for 15 min to collect the upper clear serum. The serum was diluted 20 times with PBS and serum SAA3 levels were determined by ELISA (EIAab) according to the manufacturer's protocol.

### Total Cholesterol, HDL‐C, and LDL‐C Assay

Blood was collected using an RNA‐free EP tube and centrifuged at room temperature at 3000 RPM for 15 min to collect the upper clear serum. Serum total cholesterol, HDL‐C, and LDL‐C levels were determined by corresponding kit (Nanjing Jiancheng Biological Engineering Research Institute) according to the manufacturer's protocol.

## Conflict of Interest

The authors declare no conflict of interest.

## Author Contributions

B.G., X.B., and Y.Z. conceived the project. S.H., Y.J., and J.L. performed the majority of the experiments and analyzed data. L.M. and S.Z. constructed the SAA3 knockout mouse strain. Z.Q. performed analyses on RNA‐seq data. Y.J., Y.L., W.L., Z.X., and W.Z. helped with feeding the mice and performed some of the immunohistochemistry and immunofluorescence experiments. B.G. and Y.Z. provided helpful discussions and methodology, and guided experiments and data analysis. B.G. and X.B. wrote the manuscript.

## Supporting information

Supporting Information

Supporting Information

## Data Availability

Research data are not shared.
