# Supplementary material for: Osteocytes/Osteoblasts Produce SAA3 to Regulate Hepatic Metabolism of Cholesterol
Source: Adv Sci (Weinh). 2024 Apr 13;11(24):2307818. doi: 10.1002/advs.202307818 (PMC11199997; doi:10.1002/advs.202307818)
Supplement: Supplementary file 1 — Supporting Information [file ADVS-11-2307818-s002.pdf]

ADVANCED SCIENCE
Open Access

Supporting Information

for *Adv. Sci.*, DOI 10.1002/advs.202307818

Osteocytes/Osteoblasts Produce SAA3 to Regulate Hepatic Metabolism of Cholesterol

Shijiang Huang, Yuanjun Jiang, Jing Li, Linlin Mao, Zeyou Qiu, Sheng Zhang, Yuhui Jiang, Yong Liu, Wen Liu, Zhi Xiong, Wuju Zhang, Xiaolin Liu, Yue Zhang, Xiaochun Bai* and Bin Guo**

Supporting Information for

Osteocytes/osteoblasts produce SAA3 to regulate hepatic metabolism of cholesterol.

Shijiang Huang[1,8], Yuanjun Jiang[1,8], Jing Li[2,8], Linlin Mao[1], Zeyou Qiu[3,4], Sheng Zhang[1], Yuhui Jiang[1], Yong Liu[1], Wen Liu[1], Zhi Xiong[1], Wuju Zhang[1,5], Xiaolin Liu[1], Yue Zhang[1,*], Xiaochun Bai[1,6,*], Bin Guo[1,7,9,*]

[1] State Key Laboratory of Organ Failure Research, Department of Cell Biology, School of Basic Medical Sciences, Southern Medical University, Guangzhou, Guangdong, 510515, China

[2] Department of Obstetrics and Gynecology, Nanfang Hospital, Southern Medical University, Guangzhou, Guangdong, 510515, China

[3] Department of Biochemistry and Molecular Biology, School of Basic Medical Sciences, Southern Medical University, Guangzhou, Guangdong, 510515, China

[4] Equipment material department, West China Xiamen Hospital of Sichuan University, Xiamen, Fujian, 361000, China

[5] Central Laboratory, The Fifth Affiliated Hospital, Southern Medical University, Guangzhou, Guangdong, 510900, China

[6] Guangdong Provincial Key Laboratory of Bone and Joint Degenerative Diseases, The Third Affiliated Hospital of Southern Medical University, Guangzhou, Guangdong, 510630, China

[7] The Tenth Affiliated Hospital, Southern Medical University, Dongguan, Guangdong, 523018, China

[8] These authors contributed equally

[9] Lead Contact

[*]Correspondence: Bin Guo (bzg18@smu.edu.cn), Xiaochun Bai (baixc15@smu.edu.cn), and Yue Zhang (yugi@smu.edu.cn)

This PDF file includes:

Figure S1-S3
Table S1-S2

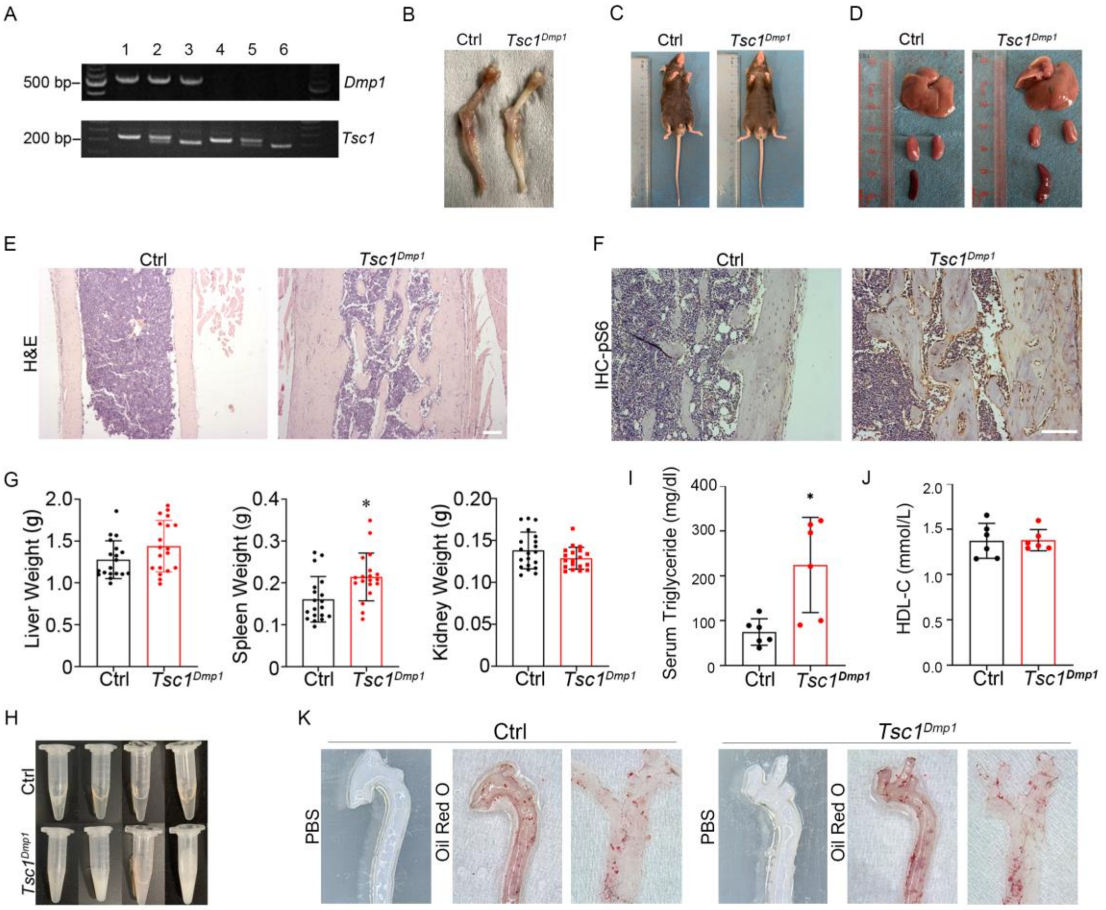

Figure S1 Characteristics of $Tsc1^{Dmp1}$ mice. (A) Representative PCR products are resolved on a 2% agarose gel for genotype test, with each lane representing DNA harvested from tail biopsy from mouse from indicated strain. Top: *Dmp1-Cre* detection; bottom: *Tsc1-LoxP* detection. Lane 1: $Dmp1^{Cre}$ $Tsc1^{fl/fl}$; Lane 2: $Dmp1^{Cre}$ $Tsc1^{fl/+}$; Lane 3: $Dmp1^{Cre}$; Lane 4: $Tsc1^{fl/fl}$; Lane 5: $Tsc1^{fl/+}$; Lane 6: wild type. (B) Representative gross view of lower limb bone collected from 6-month-old Ctrl and $Tsc1^{Dmp1}$ mice. (C) Representative overall view of Ctrl and $Tsc1^{Dmp1}$ mice at 6 months of age. (D) Representative gross view of internal organs of Ctrl and $Tsc1^{Dmp1}$ mice at 6 months of age. (E) Representative H&E staining results of the mid-femur of 6-month-old Ctrl and $Tsc1^{Dmp1}$ mice. Scale bar, 200 μm. (F) Representative immunohistochemical staining for pS6 expression in mid-femur of 6-month-old Ctrl and $Tsc1^{Dmp1}$ mice. Scale bar, 200 μm. (G) Weight statistics of liver, spleen, and kidney collected from $Tsc1^{Dmp1}$ mice and control littermates at 6 months of age (n = 19, 11 females, 8 males). (H) Appearance of serum samples collected from Ctrl and $Tsc1^{Dmp1}$ mice at 6 months of age (n = 4, 2 females, 2 males). (I-J) Serum triglyceride and HDL-C concentrations in $Tsc1^{Dmp1}$ mice and control littermates at 6 months of age (n = 6, 4 females, 2 males). (K) Representative images of whole aorta of Ctrl and $Tsc1^{Dmp1}$ mice at the age of 13 months before (left) and after (middle) Oil Red O staining. The arterial tree stained with Oil Red O was then opened longitudinally to flatten the aorta for imaging. Images on the right show longitudinally split and pinned whole aorta (n = 3, 2 females, 1 male). Data represent mean ± SD; each symbol represents one animal. * $p < 0.05$ by unpaired t test (G, I, J).

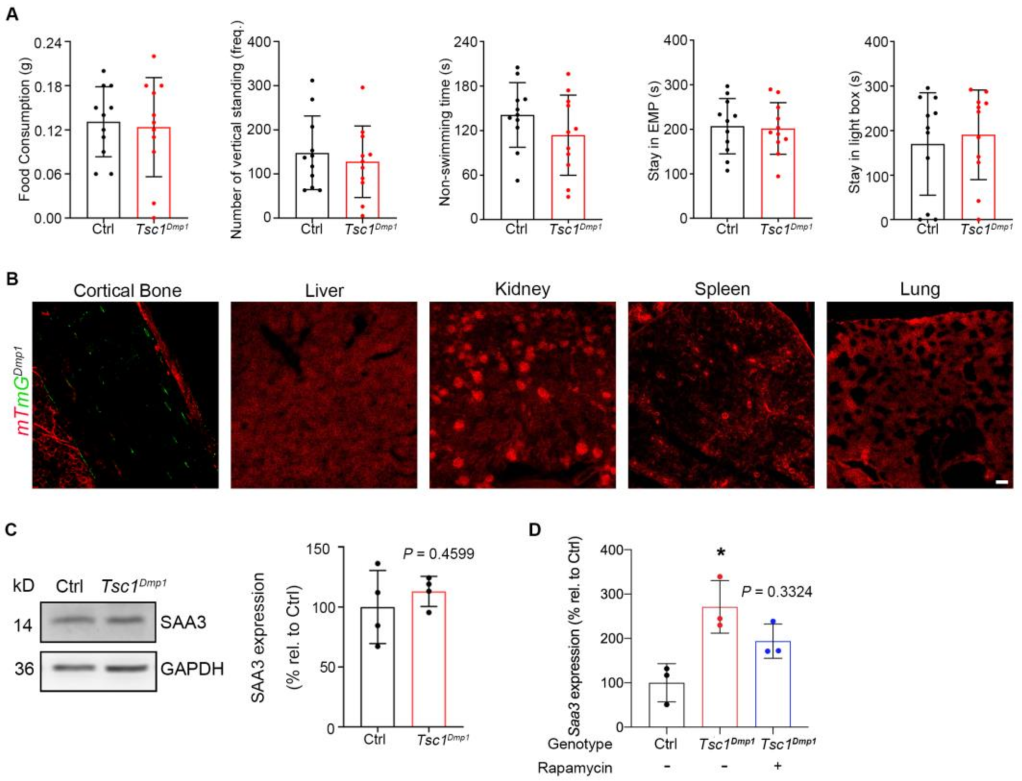

Figure S2 Characteristics of *Tsc1*^{Dmp1} mice (continued). (A) Behavioral experiments conducted with *Tsc1*^{Dmp1} mice and control littermates at 6 months of age (n = 11, 7 females, 4 males): food consumption in a novel environment, the number of times mice stood in an open field, immobility duration of mice in the forced swim test, duration of time spent in the closed arm of an elevated plus maze, and duration of time spent in the lit compartment of a light-dark box. (B) Representative images of frozen sections of cortical bones, liver, kidneys, spleen, and lung collected from *Dmp1*^{Cre}/*mTmG*$^{fl/fl}$ mice were taken under a fluorescence confocal microscope. (C) Western blot shows no significant difference in the expression of SAA3 in visceral adipose tissues between *Tsc1*^{Dmp1} mice and control littermates at 6 months of age (n = 4, 3 females, 1 male). (D) *Saa3* mRNA levels in cortical bones collected from *Tsc1*^{Dmp1} mice and control littermates treated with or without rapamycin (2 mg/kg of body weight/day for 2 weeks) at 9 months of age (n = 3, Ctrl: 2 females, 1 male; *Tsc1*^{Dmp1} without rapamycin: 2 females, 1 male; *Tsc1*^{Dmp1} with rapamycin: 2 females, 1 male). Scale bar, 100 µm. Data represent mean ± SD; each symbol represents one animal. * $p < 0.05$ by unpaired t test (A, C) or two-way ANOVA (D).

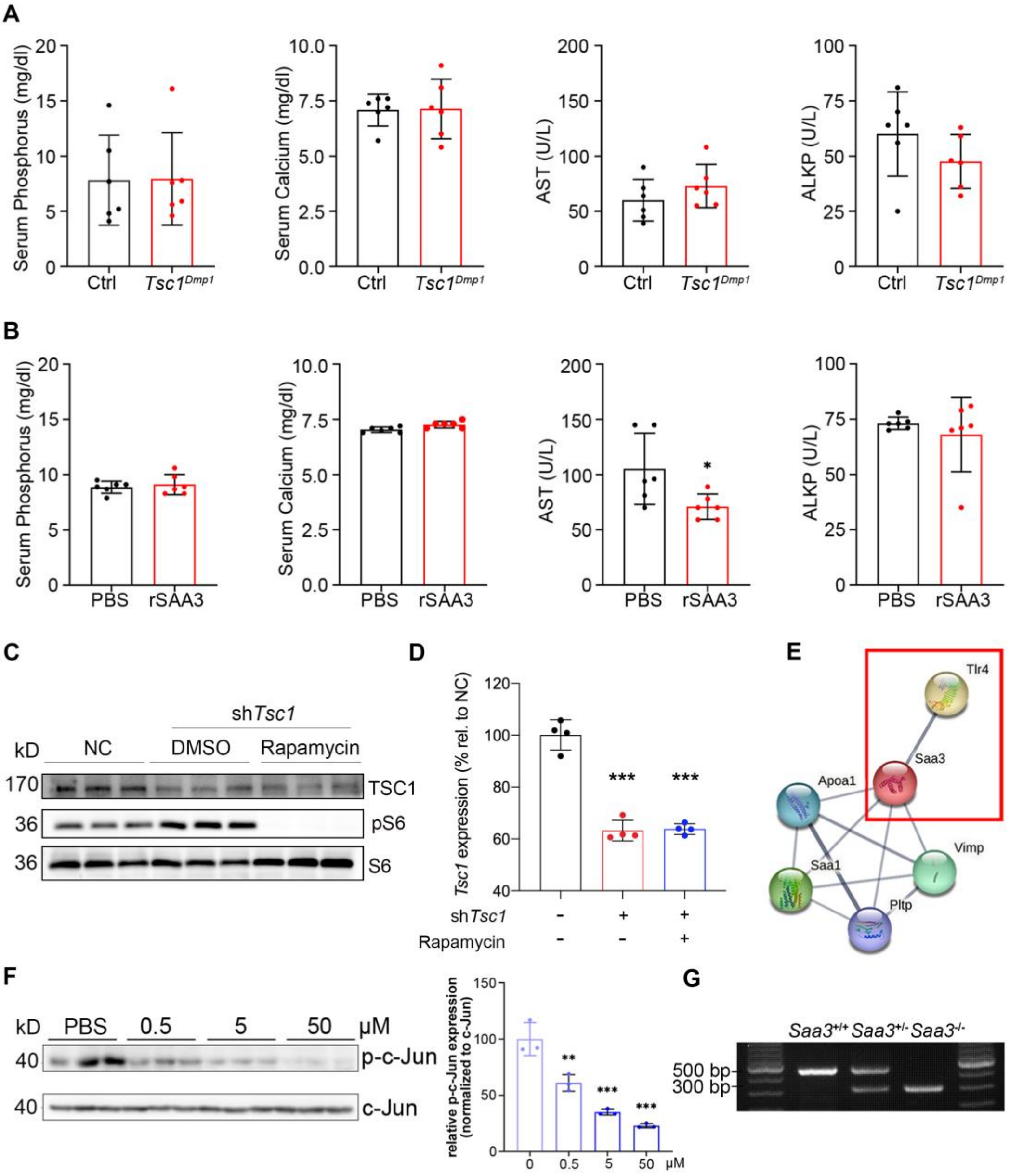

Figure S3 Characteristics of *Tsc1*^{Dmp1} mice, mice treated with rSAA3, or cultured cells underwent indicated treatments. (A) Quantification of indicated serum biochemicals in 6-month-old Ctrl and *Tsc1*^{Dmp1} mice (n = 6, 4 females, 2 males): serum phosphate, serum calcium, aspartate aminotransferase (AST), alkaline phosphatase (ALKP). (B) Quantification of indicated serum biochemicals in mice injected with PBS or rSAA3: serum phosphate, serum calcium, AST, ALKP (n = 6, 6 males). (C, D) Western blot analyses and qRT-PCR of TSC1/*Tsc1*, pS6, and S6 in Mlo-Y4 cells with indicated treatments (n = 4). (E) Predicting results from STRING database showing interacting proteins of SAA3. Outlined red box on the right shows a predicted direct interaction between TLR4 and SAA3. (F) Western blot analyses of p-c-Jun and c-Jun in Hepa1-6 cells treated with indicated concentrations of SP600125 (n = 3). (G) Genotypes of *Saa3*$^{-/-}$ mice were identified by the agarose gel electrophoresis. Data represent mean ± SD; each symbol represents one animal. * $p < 0.05$, ** $p < 0.01$, *** $p < 0.001$ by unpaired t test (A, B), one-way ANOVA (F) or two-way ANOVA (D).

Table S1 Differential expression genes (DEGs) in cortical bone of *Tsc1*^{Dmp1} mice.

See Annex 1 for details

Table S2 Key resources table.

REAGENT or RESOURCE	SOURCE	IDENTIFIER
Antibodies		
CYP7A1 Rabbit pAb	Abclonal	Cat#A10615 RRID:AB_2758151
Anti-c-Jun(S63) Antibody	HUABIO	Cat#1608-3 RRID:AB_2936843
Anti-Phospho-c-Jun Antibody	HUABIO	Cat#ET1701-32 RRID:AB_2936842
Phospho-S6 Ribosomal Protein (Ser235/236) Antibody	Cell Signaling Technology	Cat#2211S RRID:AB_331679
Ribosomal protein S6	Santa Cruz Blotechnology	Cat#sc-74459 RRID:AB_1129205
TSC1 Polyclonal Antibody	Invitrogen	Cat#PA5-80193 RRID:AB_2747307
Mouse DMP-1 Antibody	R&D Systems	Cat#AF4386 RRID:AB_2091367
SAA3 Rabbit pAb	Abclonal	Cat#A11948 RRID:AB_2758887
Rat Control IgG	Abclonal	Cat#AC034 RRID:AB_2771948
TLR4/MD-2 Complex Monoclonal Antibody	Invitrogen	Cat#14-9924-82 RRID:AB_468617
Peroxidase-conjugated AffiniPure Goat Anti-Rabbit IgG (H+L)	Jackson	Cat#111-035-003 RRID:AB_2313567
Peroxidase-conjugated AffiniPure Goat Anti-Mouse IgG (H+L)	Jackson	Cat#115-035-003 RRID:AB_10015289
Peroxidase-conjugated AffiniPure Goat Anti-Rat IgG (H+L)	Jackson	Cat#112-035-143 RRID:AB_2338138
Donkey anti-Sheep IgG (H+L) Highly Cross-Adsorbed Secondary Antibody, Alexa Fluor 594	Invitrogen	Cat#A16047 RRID:AB_2534721
Donkey anti-Mouse IgG (H+L) Highly Cross-Adsorbed Secondary Antibody, Alexa Fluor 488	Invitrogen	Cat#A21202 RRID:AB_141607
Donkey anti-Rabbit IgG (H+L) Highly Cross-Adsorbed Secondary Antibody, Alexa Fluor 488	Invitrogen	Cat#A21206 RRID:AB_2535792
Donkey anti-Mouse IgG (H+L) Highly Cross-Adsorbed Secondary Antibody,	Invitrogen	Cat#A21203 RRID:AB_141633

Alexa Fluor 594		
Donkey anti-Rabbit IgG (H+L) Highly Cross-Adsorbed Secondary Antibody, Alexa Fluor 594	Invitrogen	Cat#A21207 RRID:AB_141637
β-actin (MG3) Mouse Monoclonal Antibody	Beijing Ray Antibody Biotech	Cat#RM2001 RRID:AB_2756462
GAPDH(MC4) Mouse Monoclonal Antibody	Beijing Ray Antibody Biotech	Cat#RM2002 RRID:AB_2756459
Recombinant proteins and Chemicals		
Recombinant Mouse Saa3 protein	Signalway Antibody	Cat#AP71155
Sp600125	Selleck	Cat#S1460
Rapamycin	MedChemExpress	Cat#HY-10219
Pen Strep	Gibco	Cat#15140-122
Sodium pyruvate	Gibco	Cat#11360070
Complete Protease Inhibitor mini EASY packs EDTA-Free	Roche	Cat#05892791001
Chaps	Bio-RAD	Cat#1610460
5%BSA	BOSTER	Cat#AR0004
PageRuler™Prestained Protein Ladder	Thermo Fisher Scientific	Cat#26616
TRIzol	Thermo Fisher Scientific	Cat#15596018
Fetal Bovine Serum（Prime）	Excellbio	Cat#FSP500
BI FBS, US origin	Biological Industries	Cat#04-400-1A
Nuclease-Free Water	Promega	Cat#P1193
Paraformaldehyde	Macklin	Cat#P804537
HiScript II Q RT SuperMix for qPCR (+gDNA wiper)	Vazyme	Cat#R223-01
ChamQ SYBR qPCR Master Mix (High ROX Premixed)	Vazyme	Cat#Q341-02
2xTaq Plus Master Mix II(Dye Plus)	Vazyme	Cat#P213-02
cOmplete ULTRA Tablets	Roche	Cat#5892791001
Agar	Sangon Biotech	Cat#A100637
Percoll	Solarbio	Cat#P8370
DAB	ZSGB-BIO	Cat#ZLI-9018
Collagenase V	Sigma	Cat#C9263
DAPI	Sigma	Cat#D9542
rProtein A/G Plus Magpoly Beads	Abclonal	Cat#RM09008
RIPA lysis buffer	Leagene	Cat#PS0013
Lilie-Mayer hematoxylin staining solution	Leagene	Cat#DH0001
Modified Oil Red O Staining Kit	Beyotimes	Cat#C0157S
Critical commercial assays		
MEGAshortscript™ T7 Kit	Thermo Fisher Scientific	Cat#AM1354
mirVana™ miRNA Isolation Kit, without phenol	Thermo Fisher Scientific	Cat#AM1561

Mouse Total Bile Acids Assay Kit	Crystal Chem	Cat#80471
CATALYST AST	IDEXX	Cat#98-11069-01
CATALYST ALKP	IDEXX	Cat#98-11066-01
CATALYST CA	IDEXX	Cat#98-11071-01
CATALYST PHOS	IDEXX	Cat#98-11083-01
Total cholesterol assay kit	Nanjing Jiancheng Biological Engineering Research Institute	Cat#A111
Low-density lipoprotein cholesterol assay kit	Nanjing Jiancheng Biological Engineering Research Institute	Cat#A113
High-density lipoprotein cholesterol assay kit	Nanjing Jiancheng Biological Engineering Research Institute	Cat#A112
Triglyceride assay kit	Nanjing Jiancheng Biological Engineering Research Institute	Cat#A110
Mouse SAA/SAA1 PicoKine ELISA Kit	Boster	Cat#EK1190
ELISA Kit FOR Serum amyloid A-3 protein	EIAab	Cat#E1795m
Western Lightning Plus ECL	Perkinelmer	Cat#0RT2655
BCA protein content detection kit	KeyGEN BioTECH	Cat#KGP902
Experimental models: Cell lines		
Hepa1-6	ATCC	CRL-1830
Mlo-Y4	EIAab	iCell-m037
mHepatocyte	This paper	N/A
mKupffer cells	This paper	N/A
Experimental models: Organisms/strains		
Tsc1 $^{fl/fl}$ mice	The Jackson Laboratory	Stock No: 005680 RRID:IMSR_JAX:005680
Dmp1-Cre mice	The Jackson Laboratory	Stock No:023047 RRID:IMSR_JAX:023047
B6.129(Cg)-Gt(ROSA)26Sor$^{tm4(ACTB-tdTomato,-EGFP)Luo}$/J mice	The Jackson Laboratory	Stock No:007676 RRID:IMSR_JAX:007676
Saa3-KO mice	This paper	N/A
Oligonucleotides		
Tsc1-Flox(F)-genotyping primer GTCACGACCGTAGGAGAAGC	The Jackson Laboratory	N/A
Tsc1-Flox(R)-genotyping primer GAATCAACCCCACAGAGCAT	The Jackson Laboratory	N/A
Dmp1-Cre(F)-genotyping primer CCCGCAGAACCTGAAGATG	The Jackson Laboratory	N/A

Dmp1-Cre(R)-genotyping primer GACCCGGCAAAACAGGTAG	The Jackson Laboratory	N/A
Saa3-sgRNA-F CTTCATCCTGCTATAGGGCC	This paper	N/A
Saa3-sgRNA-R GTCATCAGGTAACACGGGTC	This paper	N/A
Saa3-F1-genotyping primer TGAAATCCAGTGGGGTTTGTTACCAG	This paper	N/A
Saa3-F2-genotyping primer CATTTCTGTGGGTTTTGGGAAAAGCT	This paper	N/A
Saa3-R-genotyping primer CTGTCAGCCTCACACTGGATGAG	This paper	N/A
Gapdh-RT-F AGGTCGGTGTGAACGGATTTG	This paper	N/A
Gapdh-RT-R TGTAGACCATGTAGTTGAGGTCA	This paper	N/A
Saa1-RT-F TGAAGGAAGCTAACTGGAAAAACTC	This paper	N/A
Saa1-RT-R CACTGATTTTCTCAGCAGCCC	This paper	N/A
Saa2-RT-F TTCTTCGGCAGAGGACACG	This paper	N/A
Saa2-RT-R CCCAACACAGCCTTCTGAACTAA	This paper	N/A
Saa3-RT-F AATACTTCCATGCTCGGGGG	This paper	N/A
Saa3-RT-R GCTCCATGTCCCGTGAACTT	This paper	N/A
Cyp7a1-RT-F CACCATTCCTGCAACCTTCTGG	This paper	N/A
Cyp7a1-RT-R ATGGCATTCCCTCCAGAGCTGA	This paper	N/A
Alb-RT-F CAGTGTTGTGCAGAGGCTGACA	This paper	N/A
Alb-RT-R GGAGCACTTCATTCTCTGACGG	This paper	N/A

Clec4f-RT-F TCACAGCCTTGGAGACCTGAGT	This paper	N/A
Clec4f-RT-R CCTAAGCCTCTGGATAGCCACT	This paper	N/A
Software		
FV10-ASW 3.1	Olympus	http://www.olympus.com.cn/
ZEN 2011	Zeiss	https://www.zeiss.com/microscopy/int/products/microscope-software/zen.html
Image J	Image J	https://imagej.nih.gov/ij/
GraphPad Prism 8.0	Prism	http://www.graphpad-prism.cn/prism.html
Adobe Photoshop 6.0	Adobe	https://www.adobe.com/cn/products/photoshop.html
